# A Rare Case of Posteriorly Migrated Sequestered Lumbar Disc Herniation Through the Interlaminar Space

**DOI:** 10.3390/reports8030169

**Published:** 2025-09-03

**Authors:** Merih Can Yilmaz, Keramettin Aydin

**Affiliations:** Department of Neurosurgery, VM Medical Park Hospital, 55200 Samsun, Turkey

**Keywords:** lumbar disc herniation, sequestrated disc, interlaminar

## Abstract

**Background and Clinical Significance**: Posteriorly migrated lumbar disc herniation [PMLDH] is a rare entity that may present with atypical clinical and radiological features, often mimicking other spinal pathologies. Migration of sequestered fragments through the interlaminar space is exceptionally uncommon, and diagnostic challenges are further amplified in the presence of spinal instability. While MRI and CT are generally sufficient for diagnosis, undetected lesions on preoperative imaging may complicate clinical management. **Case Presentation**: A 59-year-old male presented with acute low back pain and left-sided radiculopathy. Examination revealed mild motor weakness in ankle dorsiflexion. MRI showed L4–L5 segmental instability with central canal stenosis but no migrated disc fragment. Owing to neurological deficit, decompressive laminectomy with posterior instrumentation was performed. Intraoperatively, a posteriorly migrated sequestered fragment compressing the thecal sac was excised and confirmed as degenerative disc material. Postoperatively, the patient’s neurological deficit and radicular pain resolved, with no new complaints at 3-month follow-up. **Conclusions**: This case highlights an unusual presentation of PMLDH in a patient with lumbar stenosis and spinal instability, undetected on preoperative imaging. Recognition of the biomechanical predisposition at the L3–4 and L4–5 levels is important in understanding such rare migrations. Although literature emphasizes early surgical intervention for PMLDH, our patient required urgent surgery due to neurological deficits rather than a definitive preoperative diagnosis. Further studies are warranted to clarify the relationship between instability and posterior migration.

## 1. Introduction and Clinical Significance

Lumbar disc herniation is one of the most common spinal disorders, affecting millions of individuals worldwide [1]. It typically presents with low back pain, radiculopathy, and sometimes with more severe neurological deficits such as cauda equina syndrome [2]. The pathophysiology of lumbar disc herniation involves the displacement of intervertebral disc material, which may either remain contained within the disc space or herniate into the surrounding epidural space. In the majority of cases, herniated disc fragments migrate anteriorly or laterally, following the natural anatomy of the spine [3,4]. However, posterior migration of sequestered disc fragments is a rare occurrence [5], with even rarer instances of such fragments migrating through the interlaminar space.

The interlaminar space, located between the posterior elements of adjacent vertebrae, is a narrow anatomical region typically protecting the spinal cord and nerve roots. Migration of disc material through this space poses significant diagnostic challenges, as it often results in unusual radiological and clinical presentations that can be confused with other pathologies such as epidural hematoma, spinal tumours, or abscesses [6]. This atypical migration is even more concerning in patients with underlying spinal instability, as the altered biomechanics of the spine may increase the likelihood of such unusual disc fragment displacements [7].

The occurrence of a sequestered lumbar disc fragment migrating posteriorly through the interlaminar space can result in compression of the thecal sac, potentially causing significant neurological deficits, including radicular pain, motor weakness, or even paralysis, depending on the location and size of the herniated fragment [5]. Conventional imaging modalities such as MRI and CT are generally adequate for diagnosing posteriorly migrated lumbar disc herniation (PMLDH) [8]. However, as in our case, management and treatment become challenging when preoperative radiological findings fail to demonstrate the lesion.

This case report describes the posterior migration of PMLDH and sequestrated material through the interlaminar space in a patient with lumbar stenosis and spinal instability, which could not be detected on preoperative radiological imaging. This uncommon condition is discussed in the context of the existing literature, with emphasis on the diagnostic challenges and therapeutic considerations.

## 2. Case Presentation

A 59-year-old male patient presented to our hospital with acute onset of low back pain and radicular symptoms radiating to the left lower extremity. Neurological assessment demonstrated mild motor weakness in left ankle dorsiflexion graded 4/5 on the MRC scale. Patellar and achilles reflexes were preserved, and no sensory impairment was observed. This finding was considered clinically significant as it indicated nerve root compression. The Visual Analog Scale [VAS] score was determined to be 8, indicating a significant loss of quality of life.

**Figure 1:**
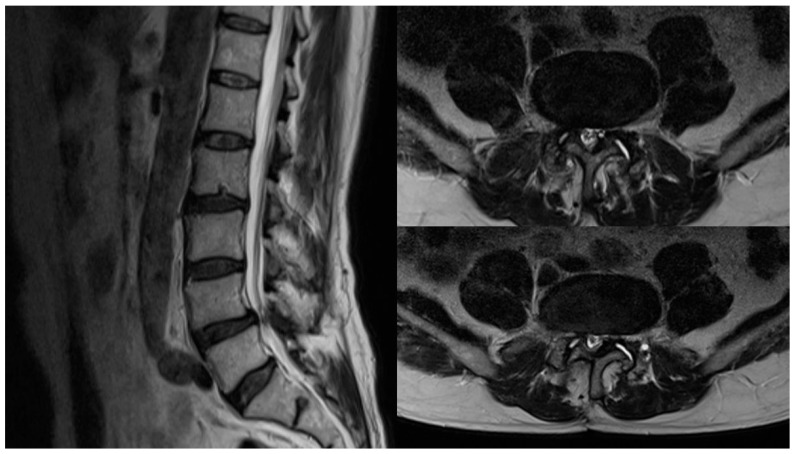
Sagittal and axial lumbar MRI images of the patient in the preoperative period [Axial images demonstrate the L4–L5 intervertebral disc space].

No pathological findings were detected in routine laboratory tests. The patient’s medical history was unremarkable. Lumbosacral MRI revealed segmental instability due to facet joint degeneration and loss of disc space, along with marked central spinal canal stenosis at the L4–5 intervertebral disc level (Figure 1).

However, no structures consistent with typical interlaminar or migration patterns of sequestrated disc material were observed on imaging. In view of the neurological motor deficit, along with L4–L5 segmental instability and canal stenosis, decompressive laminectomy with posterior segmental instrumentation at L4–L5 was planned. The patient received detailed counselling regarding the procedure, and written informed consent was obtained.

**Figure 2:**
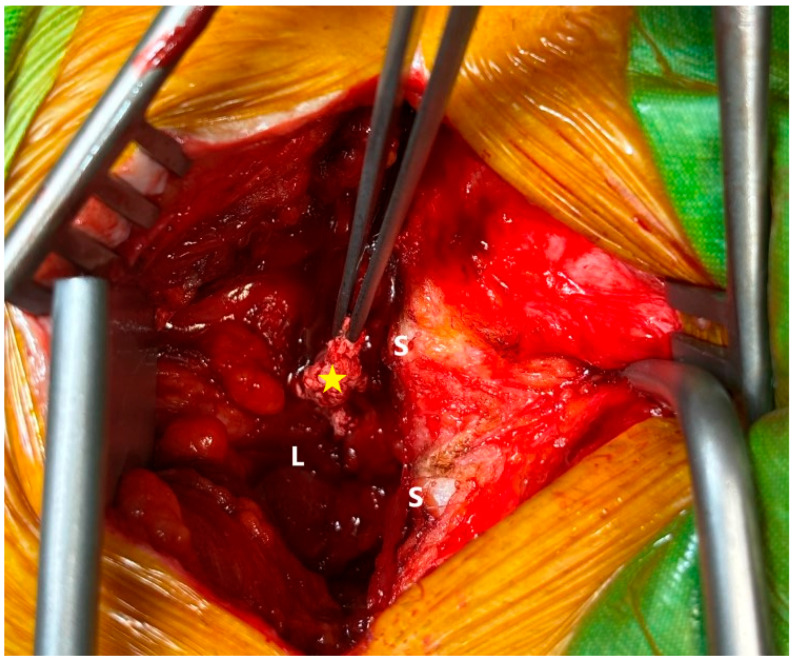
Intraoperative interlaminar space with sequestrated disc herniation [S: spinous process, L: lamina, yellow star: sequestered fragment at the interlaminar level].

During the planned surgery, following dissection of the posterior lumbar musculature and exposure of the lamina, a fragment migrating posteriorly through the interlaminar space was identified (Figure 2).

The fragment extended from the interlaminar region into the posterior epidural space and was found to compress the thecal sac overlying the dura mater. The specimen was excised and submitted for histopathological examination. Following decompressive laminectomy, the operation was finalized with transpedicular segmental stabilization at L4 and L5. The patient’s pathological examination revealed degenerative disc material.

During the postoperative period, the patient’s motor deficit and radicular pain were completely resolved [VAS score: 0]. The patient was discharged from the hospital without any additional complications during their hospital stay. At the 3-month outpatient follow-up, the patient reported no new complaints.

## 3. Discussion

The prevalence of lumbar disc herniation in adults is around 5% and continues to increase [9]. The sequestrated disc herniation subtype has the lowest incidence of disc herniation. In cases of sequestered or extruded disc herniations, the disc fragment typically shifts to the right or left side of the anterior epidural space in 94% of cases. It crosses the midline and, on rare occasions, extends into the posterior epidural space [10].

The literature review implies that patients with the sequestration subtype of disc herniation are significantly more likely to show spontaneous regression than those with bulging or protruding discs. In the absence of definitive surgical indications, conservative treatment is preferred in patients with large lumbar disc herniations, especially if they are of the extrusion or sequestration subtype [11].

Sucuoglu et al. reported that most patients with sequestered lumbar disc herniation experienced spontaneous regression within six months following MRI. Although patients who underwent early surgery showed greater improvement in pain and disability scores, the differences were not statistically significant when compared to those who had spontaneous regression at six months [12]. However, in situations involving neurological deficits, such as cauda equina syndrome, prompt and precise diagnosis is essential to prevent additional neurological deterioration. Early surgical intervention to excise sequestered disc fragments in patients with neurological symptoms has demonstrated outstanding functional and clinical outcomes [13,14]. In our case, although no sequestered fragment was observed on MRI images, the patient’s neurologic deficit necessitated surgery.

It has been noted that factors such as ligament insufficiency and other structures that anchor fragments to the anterior epidural space, along with larger spinal canals that provide more space between nerve roots, thecal sac, and intervertebral discs, may contribute to the displacement of sequestered fragments into the posterior epidural space [15].

Although posteriorly migrated lumbar disc herniations (PMLDH) are uncommon, a systematic review addressing this rare entity has been reported in the literature. The majority of affected patients were male (80%), with a mean age of 54 years [8]. Clinical manifestations primarily consisted of cauda equina syndrome, radicular symptoms, and lumbar pain [5]. In the majority of reported cases, posterior migration occurred at the L3–L4 segment (75%) and less frequently at the L4–L5 segment (25%) [16].

The literature has been reviewed to clarify the factors associated with the predominance of PMLDH at the L3–L4 level, the median age of 54 years, and the higher incidence in males. Findings suggest that lumbar disc degeneration is more often observed at the lower levels (L4–L5, L5–S1) in younger patients, whereas degeneration at the upper levels (L1–L2, L2–L3, L3–L4) is more common in older individuals [17]. At the L3–L4 segment, the spinal canal is relatively wider and the intervertebral disc is oriented more horizontally. These anatomical characteristics, in conjunction with the course of the nerve root, may facilitate epidural migration [16]. Age-related biomechanical studies have demonstrated that, in flexed sitting and standing postures, the mechanical load transmitted to L3 and L4 corresponds to approximately 2.5 times the total body weight [7]. Evidence also indicates a notable relationship between high levels of physical activity and the development of disc degeneration [18,19]. PMLDH occurs more frequently in males than in females. Reviews of the literature indicate that women have a smaller cross-sectional area of trunk core musculature compared to men, and that the orientation of muscle forces also differs between the sexes [20,21]. Spinal range of motion also demonstrates sex-related differences; males typically exhibit greater lumbar extension, whereas females show increased lateral flexion or side bending [22]. Women also have significantly greater lumbar lordosis than men [23]. This suggests that men may generate higher thrust forces, thereby facilitating the migration of disc fragments into the posterior epidural space.

Although the majority of PMLDH cases are identified by MRI (80–93%) and/or CT imaging (7–11%), myelography (11.5%) has also been utilized for diagnosis, especially in earlier reports [24,25,26,27]. Myelography primarily demonstrates the mass effect and dural sac compression, but offers no insight into the underlying characteristics of the lesion [5]. Contrast-enhanced MRI represents the most reliable diagnostic modality [24,28,29]. In PMLDH, disc fragments typically present as hypointense on T1-weighted sequences, hyperintense on approximately 80% of T2-weighted sequences, and display variable signal characteristics in the remaining 20% [30]. On short-time inversion recovery (STIR) imaging, the fragments usually appear hyperintense, reflecting increased local vascular perfusion [29].

In the differential diagnosis, synovial cysts, epidural abscesses, and hematomas should be taken into account, while neoplastic pathologies (such as meningioma, metastasis, lipoma, lymphoma, and hemangioma) and postoperative fibrosis may also present with imaging characteristics resembling PMLDH [6,31,32,33,34].

Conservative management constitutes approximately 4–5% of reported PMLDH cases. In the absence of progressive neurological impairment or cauda equina syndrome, non-surgical treatment is recommended for all patients. Commonly applied modalities include analgesics, corticosteroids, and physiotherapy [5,8].

Surgical treatment was applied to 95% of PMLDH cases [5,8]. Multiple investigators have advocated early surgical intervention as the primary therapeutic approach in cases with large posteriorly migrated sequestered disc fragments, aiming to prevent serious neurological complications such as cauda equina or conus medullaris syndromes [30,35,36]. In PMLDH cases, decompressive laminectomy represents the most frequently employed surgical technique [8]. This approach provides full visualization and facilitated removal of the fragment, lowers the likelihood of inadvertent dural injury, decreases traction on neural elements, and shortens operative time—an advantage of particular significance in urgent settings [37,38]. Hemilaminectomy is the second most frequently utilized surgical technique, aiming to limit spinal instability while permitting adequate bone removal [8,16]. In the context of degenerative disc disease, spinal fusion in conjunction with laminectomy has been advocated to decrease loading stresses on the disc spaces and facet joints, while also preventing potential superimposed instability [8,34,39,40]. In appropriately selected patients, minimally invasive techniques such as the interlaminar endoscopic approach have been reported to achieve success rates comparable to those of laminectomy [41].

The main limitation of this report is that it is based on a single case, which restricts the generalizability of the findings. Further studies with larger patient cohorts are needed to better clarify the association between instability and posterior migration.

## 4. Conclusions

This case report describes a rare instance of sequestered disc herniation migrating from the posterior epidural space into the interlaminar region. Although the existing literature does not provide a definitive explanation for the underlying etiology, it highlights the biomechanical characteristics of the L3–4 and L4–5 levels and their predisposition to pathological changes in the presence of instability. Nevertheless, further comprehensive studies are required to substantiate the association between posterior migration and segmental instability. Moreover, while prior reports stress the role of early surgical intervention in PMLDH, in our case, early surgery was performed not based on a preoperative diagnosis of PMLDH, but due to the presence of neurological deficits.

## Data Availability

The original data presented in this study are available on reasonable request from the corresponding author. The data are not publicly available due to privacy concerns.

## Abbreviations

The following abbreviations are used in this manuscript:

MRI	Magnetic resonance imaging
CT	Computed tomography
MRC	Medical Research Council
PMLDH	Posteriorly migrated lumbar disc herniation
VAS	Visual analog scale

## References

[1] Buser Z., Chung A.S., Abedi A., Wang J.C. (2019). The future of disc surgery and regeneration. Int. Orthop..

[2] Ray-Offor O., Wachukwu C., Onubiyi C. (2016). Intervertebral disc herniation: Prevalence and association with clinical diagnosis. Niger. J. Med..

[3] Mraja H.M., Kaya O., Mammadov T., Karadereler S., Hamzaoglu A., MRAJA H.M. (2022). Anterior-to-Posterior Epidural Migration of a Lumbar Disc Herniation at L1-L2: A Case Report. Cureus.

[4] Saad M.E., Obame F.L.O., El Asri A.C., Gazzaz M. (2024). Posterior migration of lumbar disc herniation: A case report. Radiol. Case Rep..

[5] Elsharkawy A.E., Hagemann A., Klassen P.D. (2019). Posterior epidural migration of herniated lumbar disc fragment: A literature review. Neurosurg. Rev..

[6] Montalvo Afonso A., Mateo Sierra O., Gil de Sagredo del Corral O.L., Vargas López A.J., González-Quarante L.H., Sola Vendrell E., Romero Martínez J. (2018). Misdiagnosis of posterior sequestered lumbar disc herniation: Report of three cases and review of the literature. Spinal Cord Ser. Cases.

[7] Schultz A., Andersson G., Haderspeck K., Örtengren R., Nordin M., Björk R. (1982). Analysis and measurement of lumbar trunk loads in tasks involving bends and twists. J. Biomech..

[8] Palmisciano P., Balasubramanian K., Scalia G., Sagoo N.S., Haider A.S., Alamer O.B., Chavda V., Chaurasia B., Deora H., Passanisi M. (2022). Posterior epidural intervertebral disc migration and sequestration: A systematic review. J. Clin. Neurosci..

[9] Komori H., Shinomiya K., Nakai O., Yamaura I., Takeda S., Furuya K. (1996). The natural history of herniated nucleus pulposus with radiculopathy. Spine.

[10] Schellinger D., Manz H., Vidic B., Patronas N., Deveikis J., Muraki A., Abdullah D. (1990). Disk fragment migration. Radiology.

[11] Hu C., Lin B., Li Z., Chen X., Gao K. (2021). Spontaneous regression of a large sequestered lumbar disc herniation: A case report and literature review. J. Int. Med. Res..

[12] Sucuoğlu H., Barut A.Y. (2021). Clinical and radiological follow-up results of patients with sequestered lumbar disc herniation: A prospective cohort study. Med. Princ. Pract..

[13] Hamid S., Kropuenske M., Zahran S., Alimohammadi E. (2025). Posterior epidural migration of thoracic and lumbar disc material: A comprehensive 63-year systematic review with anatomical perspectives. Neurosurg. Rev..

[14] Tatli M., Güzel A., Cevi˙ z A., Karadağ Ö. (2005). Posterior epidural migration of sequestered lumbar disc fragment causing cauda equina syndrome. Br. J. Neurosurg..

[15] Kuzeyli K., Çakr E., Usul H., Baykal S., Yazar U., Karaarslan G., Arslan E., Peksoylu B. (2003). Posterior epidural migration of lumbar disc fragments: Report of three cases. Spine.

[16] Sengoz A., Kotil K., Tasdemiroglu E. (2011). Posterior epidural migration of herniated lumbar disc fragment. J. Neurosurg. Spine.

[17] Martin M.D., Boxell C.M., Malone D.G. (2002). Pathophysiology of lumbar disc degeneration: A review of the literature. Neurosurg. Focus.

[18] Vernon-Roberts B., Moore R.J., Fraser R.D. (2007). The natural history of age-related disc degeneration: The pathology and sequelae of tears. Spine.

[19] Battié M.C., Videman T. (2006). Lumbar disc degeneration: Epidemiology and genetics. J. Bone Jt. Surg..

[20] Brinckmann P., Hoefert H., Jongen H.T. (1981). Sex differences in the skeletal geometry of the human pelvis and hip joint. J. Biomech..

[21] Marras W., Jorgensen M., Granata K., Wiand B. (2001). Female and male trunk geometry: Size and prediction of the spine loading trunk muscles derived from MRI. Clin. Biomech..

[22] Reese N.B., Bandy W.D. (2016). Joint Range of Motion and Muscle Length.

[23] Norton B.J., Sahrmann S.A., Van Dillen L.R. (2004). Differences in measurements of lumbar curvature related to gender and low back pain. J. Orthop. Sports Phys. Ther..

[24] Haddadi K., Qazvini H.R.G. (2016). Posterior epidural migration of a sequestrated lumbar disk fragment causing cauda equina syndrome in an old patient: A case report. Clin. Med. Insights Case Rep..

[25] Elgamri A., Sami A., Aqqad A., Hilmani S., Ibahioin K., Naja A., El Kamar A., El Azhari A. (2009). Posterior migration of a lumbar disc herniation as a cause of cauda equina syndrome. J. Radiol..

[26] Neugroschl C., Kehrli P., Gigaud M., Ragragui O., Maitrot D., Manelfe C., Dietemann J. (1999). Posterior extradural migration of extruded thoracic and lumbar disc fragments: Role of MRI. Neuroradiology.

[27] Lutz J., Smith R., Jones H. (1990). CT myelography of a fragment of a lumbar disk sequestered posterior to the thecal sac. Am. J. Neuroradiol..

[28] Elsharkawy A.E., Gafumbegete E., Klassen P.D. (2018). Posterior epidural migration of extruded lumbar disc fragment mimicking epidural mass: A case report. Interdiscip. Neurosurg..

[29] Chen C., Chuang Y., Yao M.S., Chiu W.-T., Chen C.-L., Chan W.P. (2006). Posterior epidural migration of a sequestrated lumbar disk fragment: MR imaging findings. Am. J. Neuroradiol..

[30] Khan Z., Sharafat S., Ali M., Haidar A., Khanzada K., Siddique M. (2013). Posterior epidural migration of herniated lumbar disc fragment: Experience with 11 cases. Gomal J. Med. Sci..

[31] El Khamary S.M., Alorainy I.A. (2006). Case 100: Spinal epidural meningioma. Radiology.

[32] Lee J.W., Cho E.Y., Hong S., Chung H., Kim J., Chang K.-H., Choi J.-Y., Yeom J.-S., Kang H.S. (2007). Spinal epidural hemangiomas: Various types of MR imaging features with histopathologic correlation. Am. J. Neuroradiol..

[33] Park T., Lee H.J., Kim J.S., Nam K. (2018). Posterior epidural disc fragment masquerading as spinal tumor: Review of the literature. J. Back Musculoskelet. Rehabil..

[34] Yoo Y.S., Ju C.I., Kim S.W., Kim D.M. (2015). Posterior epidural migration of an extruded lumbar disc mimicking a facet cyst: A case report. Korean J. Spine.

[35] Deora H., Prabhuraj A., Pruthi N. (2017). Posterior epidural migration of lumbar disc: Will the real “disc” please stand up?. Surg. Neurol. Int..

[36] Oh Y., Eun J. (2021). Posterior epidural migration of lumbar disc fragment: Case reports and literature review. Medicine.

[37] Ba M.C., Kleib R., Sy C., Diabang J., Ndoye N., Thiam A.B., Thioub M., Tine I., Badiane S.B. (2013). Lumbar disc hernia migrating to the epidural posterior space: A rare entity. Internet J. Neurosurg..

[38] Frati A., Pesce A., Palmieri M., Vangelista T., Caruso R., Salvati M., Raco A. (2017). Anterior-to-Posterior Migration of a Lumbar Disc Sequestration: Surgical Remarks and Technical Notes about a Tailored Microsurgical Discectomy. Case Rep. Surg..

[39] Moore R.J., Vernon-Roberts B., Fraser R.D., Osti O.L., Schembri M. (1996). The origin and fate of herniated lumbar intervertebral disc tissue. Spine.

[40] Li K., Li Z., Geng W., Wang C., Ma J. (2016). Postdural disc herniation at L5/S1 level mimicking an extradural spinal tumor. Eur. Spine J..

[41] Tarukado K., Tono O., Doi T. (2014). Ordinary disc herniation changing into posterior epidural migration of lumbar disc fragments confirmed by magnetic resonance imaging: A case report of a successful endoscopic treatment. Asian Spine J..

